# Urinary phthalate exposures and risk of breast cancer: the Multiethnic Cohort study

**DOI:** 10.1186/s13058-021-01419-6

**Published:** 2021-04-06

**Authors:** Anna H. Wu, Adrian A. Franke, Lynne R. Wilkens, Chiuchen Tseng, Shannon M. Conroy, Yuqing Li, Linda M. Polfus, Mindy De Rouen, Christian Caberto, Christopher Haiman, Daniel O. Stram, Loïc Le Marchand, Iona Cheng

**Affiliations:** 1 Department of Preventive Medicine, University of Southern California Keck School of Medicine, 1441 Eastlake Avenue, Rm 4443, Los Angeles, CA 90089 USA; 2 University of Hawaii Cancer Center, Honolulu, HI USA; 3 Department of Public Health Sciences, School of Medicine, University of California, Davis, Davis, CA USA; 4 Department of Epidemiology and Biostatistics, University of California, San Francisco, San Francisco, CA USA; 5 Center for Genetic Epidemiology, University of Southern California, Los Angeles, CA USA

**Keywords:** Breast cancer, Urinary phthalates, Whites, Nonwhites, Hormone receptor status, Waist-hip ratio

## Abstract

**Background:**

The epidemiologic evidence from observational studies on breast cancer risk and phthalates, endocrine disrupting chemicals, has been inconsistent. In the only previous study based on pre-diagnostic urinary phthalates and risk of breast cancer, results were null in mostly white women.

**Methods:**

We examined the association between pre-diagnostic urinary phthalates and breast cancer in a nested case-control study within the Multiethnic Cohort (MEC) study, presenting the first data from five major racial/ethnic groups in the USA. We measured 10 phthalate metabolites and phthalic acid, using a sensitive liquid chromatography mass spectrometry assay on 1032 women with breast cancer (48 African Americans, 77 Latinos, 155 Native Hawaiians, 478 Japanese Americans, and 274 Whites) and 1030 matched controls. Conditional logistic regression was used to examine risk with individual metabolites and ratios of primary (MEHP, mono-2-ethylhexyl-phthalate) to secondary (MEHHP, mono(2-ethyl-5-hydroxyhexyl); MEOHP, mono(2-ethyl-5-oxohexy)) metabolites of di-2-ethylhexyl phthalate (DEHP), a widely used plasticizer. In addition, we investigated risk associations with high (∑HMWP) and low molecular weight (∑LMWP) phthalates, as well as total phthalates which included high and low molecular weight phthalates with phthalic acid (∑LMHMPA) or without phthalic acid in molar ratios (∑LMHM_molar_) and adjusted for creatinine and potential confounders.

**Results:**

Among all women, breast cancer risk was higher for those in tertile 2 and tertile 3 of primary to secondary metabolites of DEHP (MEHP/(MEHHP + MEOHP)) in comparison to those in tertile 1; the respective odds ratios were 1.32 (95% CI 1.04–1.68) and 1.26 (95% CI 0.96–1.66) (*P*_trend_ = 0.05). Risk among Native Hawaiian women increased with exposures to eight of ten individual phthalates and total phthalates (∑LMHMPA OR_T3 vs T1_ = 2.66, 95% CI 1.39–5.12, *P*_trend_ = 0.001). In analysis by hormone receptor (HR) status, exposure above the median of ∑LMWP was associated with an increased risk of HR-positive breast cancer (OR = 1.30, 95% CI 1.05–1.60) while above the median exposure to phthalic acid was associated with an increased risk of HR-negative breast cancer (OR_above vs below median_ = 1.59, 95% CI 1.01–2.48).

**Conclusions:**

Further investigations of suggestive associations of elevated breast cancer risk with higher ratios of primary to secondary metabolites of DEHP, and differences in risk patterns by race/ethnicity and HR status are warranted.

## Introduction

Phthalates are industrial chemicals that are present in numerous consumer products and solvents, as additives, and plasticizers [1–3], and have known endocrine disrupting properties [4]. Results from the Third National Health and Nutrition Examination Survey (NHANES III) showed ubiquitous exposure of the US population to a wide variety of phthalates [5]. Mono-ethyl phthalate (MEP), mono-n-butyl phthalate (MBP), and mono-benzyl phthalate (MBzP), metabolites of phthalates, frequently found in personal care products, were detectable in over 97% of urine samples while mono-2-ethylhexyl-phthalate (MEHP), a metabolite of di-2-ethylhexyl phthalate (DEHP), found commonly in plasticizers, food packaging, and household products, was detectable in over 75% of the samples. These high exposures were observed across all ages, in whites and nonwhites, although there were age, sex, and racial/ethnic differences that likely reflected variations in exposure patterns [5] and differences in the metabolism of phthalates [6]. Phthalates are typically metabolized by phase I hydrolysis to their respective monoesters, followed by phase II conjugation, which depending on the specific phthalates can be further metabolized via oxidation to secondary metabolites [7].

Phthalates have been implicated to influence developmental and reproductive processes and exert carcinogenic effects [1, 8–10]. However, epidemiological evidence on the association between phthalate exposure and breast cancer risk remains inconsistent. The first two case-control studies reported significantly elevated risk in relation to exposure to MEP, mono(2ethyl-5-carboxy-pentyl) phthalate (MECPP) [11] and MEHP [12], but they were small studies (75 to 233 breast cancers), and measurements were conducted in post-diagnosis samples. Urinary phthalate exposures were unrelated to risk in two larger studies (~ 400–700 breast cancers) [13, 14]; only one investigated exposures before diagnosis [14]. These studies differed in study design, sample size, inclusion of in situ and invasive breast cancers [13, 14], and race/ethnicity composition. Positive associations were reported in studies conducted in Northern Mexico [11] and Alaska (mostly Eskimos, Indians, and Aleut) [12] whereas null results were found in studies of mainly whites from the Women’s Health Initiative (WHI) [14] and the Long Island Breast Cancer Study Project (LIBCSP) [13]. Breast cancer risk was also associated with di-methyl-phthalate (DMP) exposure in a Danish prospective registry study which quantified phthalate exposure using prescription files [15].

To further investigate the role of pre-diagnostic urinary phthalates and breast cancer risk, we conducted a nested case-control in the Multiethnic Cohort (MEC) study [16] that included 274 whites and 758 nonwhites (478 Japanese Americans, 155 Native Hawaiians, 77 Latinos, and 48 African Americans) diagnosed with incident breast cancer, and individually matched control women.

## Materials and methods

### Study population

The MEC is a large prospective cohort that included 96,810 men and 118,441 women aged 45–75 years from five different racial/ethnic groups (African Americans, Latinos, Native Hawaiians, Japanese Americans, and whites) living in Hawaii and California (primarily from Los Angeles County) at enrollment between 1993 and 1996 [16]. Participants completed a baseline questionnaire which assessed demographics, lifestyle, diet, and anthropometrics, and for women, menstrual and reproductive histories and hormone therapy use. Participants were followed prospectively for diagnosis of incident breast cancer (invasive and in situ) through routine yearly linkage with the California and Hawaii statewide cancer registries and for vital status through yearly linkages to the National Death Index and state death certificate files which was through 2014 for this nested case-control study. Stage at diagnosis and estrogen/progesterone receptor (ER/PR) status were obtained from cancer registries.

In 2001–2006, a prospective biorepository was established by collecting pre-diagnostic urine and blood specimens from 67,594 MEC cohort members [17]. A short questionnaire was administered at biospecimen collection which assessed weight, use of hormone therapy and medications, and other factors. For this nested case-control study, cases (*n* = 1032) were diagnosed with incident breast cancer from 2001 through 2014 after urine collection; 22% were in situ and 78% were invasive cancers. The mean time between urine collection and breast cancer diagnosis was 5.5 years (standard deviation (SD) = 3.3). For each case, we selected one control, who was alive and free of breast cancer at the age of breast cancer diagnosis, and individually matched controls to cases on area (Hawaii or California), birth year (± 1 year), race and ethnicity (white, Native Hawaiian, African American, Latino, Japanese American), urine type (first morning from California, overnight and first morning from Hawaii), date (± 1 year) of urine collection, hours of fasting (8–10, > 10 h), and time of blood draw (± 2 h). As described previously, the overnight urine collection started between 5:00 pm and 9:00 pm (depending on participant) and included all urine passed during the night and the first morning urine sample covering a period of 12 h [18]. Controls were sampled from the representative pool of subjects with existing data on obesity-related and inflammation biomarkers and genotype array data. In total, 1030 controls were identified and each control was individually matched to one case except that one white and one Native Hawaiian control were each matched to the two cases of the same race/ethnic group. There were 30 controls (19 whites, 7 Japanese Americans, 2 Native Hawaiians, 2 Latinos, 0 African Americans) and 44 cases (14 whites, 16 Japanese Americans, 6 Native Hawaiians, 4 Latinos, 4 African Americans) who had other cancers with a mean of 6.3 years (SD 5.2) and 8.4 years (SD 5.8), before the donation of urine collection, respectively. Results were unchanged without these individuals (data not shown) and they were included in the analyses.

### Laboratory measurement of urinary phthalate metabolites

Phthalate metabolites (MEP, MMP, MBP, MiBP, MBzP, MCHP, MEHP, MEHHP, MEOHP, MECPP, MCMHP) and phthalic acid measurements were conducted at the University of Hawaii Cancer Center Analytical Biochemistry Shared Resource. Dr. Adrian Franke supervised the day-to-day activities and quality assurance and quality control of phthalate measurements using state-of-the-art sensitive isotope-dilution orbitrap-based high-resolution accurate-mass liquid chromatography mass spectrometry (LCMS) assay after enzymatic hydrolysis and liquid-liquid extraction [19, 20]. In brief, 0.1 mL urine was mixed with 0.01 mL of a mixture of isotopically labeled analyte that was used as internal standards followed by incubation with a glucuronidase/sulfatase mixture at 37 °C for 90 min, extraction with methyl tertiary butyl ether, and LCMS analysis. Our lower limits of quantitation were 0.5 ng/mL for each analyte. Individual phthalate all share phthalic acid as a common metabolite as all the phthalate diesters can be metabolized to phthalic acid [21, 22]. One metabolite (mono 2-carboxy-hexyl phthalate, MCMHP) was not measured reliably and was excluded from data analysis. Personnel were blinded to case-control status, and matched pairs of cases and controls were assayed in the same batch. Replicate samples of pooled urines (5%) were included in each of 37 batches for quality control measures and coefficients of variation (CV) were calculated. The CV% (SD/mean concentration × 100) within-batch was 26.7% for phthalic acid and was < 25% for eight of the ten metabolites with a median of 22.6% for the individual metabolites (range was 5.9% (MCHP) to 35.0% (MMP)). The larger CVs likely reflect several samples close to the lower limit of detection (LLOD). The mean CV of the non-blinded pool samples was 11.7% (SD 6.9%). All analytes were adjusted to urinary creatinine [19, 20] and are shown as micrograms per gram (μg/g) of creatinine. Analytes below the LLOD were assigned a value half of the LLOD. Eight of the ten metabolites and phthalic acid were detected at levels above the LLOD in over 92% of women; this was lower for MMP (80%) and MCHP (89%) (Supplementary Table 1). We also present the geometric mean concentration for each phthalate metabolite collected in the overnight urine samples (i.e., all were from Hawaii) and first morning urine samples (i.e., all but 16 were from Los Angeles County) (Supplementary Table 1).

### Statistical analysis

We conducted conditional logistic regression, with the matched sets as strata (1028 pairs and 2 triplets), and modeled phthalate variables as tertiles using selected cutoff points based on the distribution among all controls. Odds ratios (OR_tertiles_) and 95% confidence intervals (CIs) were the primary statistics of interest, and inference was based on the Wald test. We found no evidence of a nonlinear relationship (on the log odds scale) between phthalates and risk using restricted cubic splines (data not shown). Therefore, log-transformed phthalate variables were used as trend variables to test for dose-response relationships. Models were adjusted for potential confounders that were not matching factors (e.g., established breast cancer risk factors) via indicator variables for tertiles of propensity scores for exposure to phthalates [23, 24] in order to maximize power. Specifically, an ordinal logistic regression for tertiles of each phthalate variable was performed using the following independent variables: age at urine collection, education, number of children, age at menarche, menopausal status, body mass index (BMI) at urine collection, neighborhood socioeconomic status (nSES) [25] at urine collection, smoking, alcohol intake, and Mediterranean diet energy adjusted total score [26]. The nSES index is a composite measure created by principal component analysis (PCA) of US Census data that incorporated census block group data on education, occupation, unemployment, household income, poverty, rent, and house values [25]. PCAs were conducted separately for California and Hawaii. Results across states were similar for Eigen vectors and variance explained with a single component identified. This nSES measure was categorized into quintiles based on the nSES distribution of Los Angeles County and Hawaii block groups for California and Hawaii MEC participants, respectively. The propensity for exposure was determined for each individual as the weighted average = 1 × *ρ*_1_ + 2 × *ρ*_2_ + 2 × *ρ*_3_, where *ρ*_*i*_ is the model-based probability of exposure to tertile *i.* Heterogeneity of the associations by race/ethnicity was assessed by a global test of the interaction terms between race and the phthalate trend variable. We repeated subgroup analyses for hormone receptor-positive (HR+, ER+, or PR+) and hormone receptor-negative (HR−, ER−, and PR−) cancer, as well as BMI and use of hormone therapy at urine collection. In addition, waist-hip ratio (WHR) was obtained in a follow-up questionnaire and was available for 333 cases prior to diagnosis and 946 control women; we explored risk association results by WHR. We also conducted a sensitivity analysis by restricting to invasive cases only (*n* = 798) and by lag time between time of urine collection and breast cancer diagnosis (≤ 5 years versus > 5 years), as well as excluding 187 cases that were diagnosed within 2 years of urine collection to minimize the potential effect of pre-diagnostic breast cancer on phthalate levels. Associations with *P* < 0.05 were considered statistically significant and with 0.05 ≤ *P* < 0.10 were considered suggestive. The correlations among phthalate metabolites and phthalic acid were examined using Spearman’s rho.

**Fig. 1:**
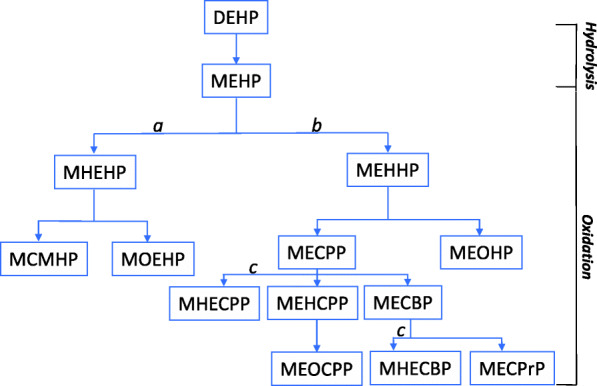
Human metabolism of di-2-ethylhexyl phthalate (DEHP), one of the most widely used phthalate plasticizers. Initial hydrolysis of this diester to mono(2-ethylhexyl) phthalate (MEHP) is followed by oxidation of the ethyl (*a*) or hexyl (*b*) side chains; both side chains are oxidized (*c*) to yield mono(1-hydroxyethyl-4-carboxybutyl) phthalate (MHECBP) and mono(1-hydroxyethyl-5-carboxypentyl) phthalate (MHECPP). The metabolites shown in the figure are MCMHP, mono(2-carboxymethylhexyl) phthalate; MECBP, mono(2-ethyl-4-carboxybutyl) phthalate; MECPP, mono(2-ethyl-5-carboxypentyl) phthalate; MECPrP, mono(2-ethyl-3-carboxypropyl) phthalate; MEHCPP, mono(2-ethyl-4-hydroxy-5-carboxypentyl) phthalate; MEHHP, mono(2-ethyl-5-hydroxyhexyl) phthalate; MEOCPP, mono(2-ethyl-4-oxo-5-carboxypentyl) phthalate; MEOHP, mono(2-ethyl-5-oxohexyl) phthalate; MHEHP, mono2-(1-hydroxyethyl) hexyl-phthalate; and MOEHP, mono2-(1-oxoethyl)-hexyl phthalate

The short-branched phthalates, DMP and DEP, are excreted in urine as the unconjugated monoesters, MMP and MEP, respectively. The longer-branched phthalates such as DEHP are hydrolyzed first to MEHP and subsequently metabolized to MEHHP, MEOHP, MECPP, and other oxidative metabolites [7, 27, 28] (Fig. 1). Oxidation of MEHP to other secondary metabolites effectively decreases the internal body burden of MEHP to presumably less toxic secondary metabolites [29, 30]. Thus, subjects with higher MEHP% and higher ratios of MEHP to major secondary DEHP metabolites (MEHHP, MEOHP, MECPP) may be at higher risk (MEHP% was calculated by converting DEHP metabolites into nanomoles (nmol) using their respective molecular weight and dividing the molar mass of MEHP by the mass of the sum of all four metabolites and then multiplying by 100). For all women, and separately in African Americans and Latinos combined, Native Hawaiians, whites, and Japanese Americans, we examined risk in relation to the 10 metabolites and phthalic acid, MEHP%, the ratios of MEHP to secondary DEHP metabolites, and summary variables, including the sum of all major DEHP metabolites (∑DEHP), low molecular weight phthalates (∑LMWP: MBP, MiBP, MEP, MMP), high molecular weight phthalates (∑HMWP: MBzP, MCHP, ∑DEHP), and total phthalate represented by the sum of all 10 phthalate metabolites and phthalic acid (∑LMHMPA) and the sum based on molar ratios of phthalate metabolites without phthalic acid (∑LMHM_molar_). We divided the concentration of each metabolite by its molecular weight to obtain the molar equivalent (micromoles/liter, μmol/L) and then summed the concentrations to get total μm/L of metabolites per creatinine unit.

## Results

The majority of participants (86%) were from Hawaii, who donated overnight (875 cases, 873 controls) or first morning urines (8 cases, 8 controls), whereas only first morning samples (149 cases, 149 controls) were collected in Los Angeles County (Table 1). Cases compared to control women were more likely to be nulliparous (15.8% vs 10.8%) and had higher BMI at urine collection (27.0 ± 5.6 vs 26.0 ± 5.6 kg/m^2^), but were otherwise comparable.

**Table 1:** Study characteristics of breast cancer cases nested within the Multiethnic Cohort (MEC)

	All cases		Controls	
	*N* = 1032	%	*N* = 1030	%
**Area**				
Hawaii	883	85.6	881	85.5
Los Angeles	149	14.4	149	14.5
**Urine type**^a^				
First morning	157	15.2	157	15.2
Overnight	875	84.8	873	84.8
**Mean age at urine collection, yrs ± SD**	66.7 ± 7.7		66.8 ±7.7	
≤ 64	487	47.2	470	45.6
65–74	376	36.4	387	37.6
75+	169	16.4	173	16.8
**Race/ethnicity**				
Japanese American	478	46.3	478	46.4
White	274	26.6	273	26.5
African American (AA)	48	4.7	49	4.8
Latino	77	7.5	76	7.4
Native Hawaiian (NH)	155	15.0	154	15.0
**Education**				
≤ High school	420	40.7	424	41.2
Some college	216	20.9	217	21.1
College graduate	192	18.6	202	19.6
Graduate school	199	19.3	180	17.5
Missing	5	0.5	7	0.7
**Age at menarche, yrs**				
< 12	555	53.8	570	55.3
13–14	370	35.9	359	34.9
> 14	100	9.7	95	9.2
Missing	7	0.7	6	0.6
**Number of children**				
Nulliparous	163	15.8	111	10.8
1 child	98	9.5	116	11.3
2–3 children	523	50.7	553	53.7
> 4 children	245	23.7	245	23.8
Missing	3	0.3	5	0.5
**Age at first live birth, yrs**				
Nulliparous	163	15.8	111	10.8
15–20	211	20.4	208	20.2
21–30	564	54.7	614	59.6
> 30	82	7.9	76	7.4
Missing	12	1.2	21	2.0
**Menopausal status**				
Premenopause	213	20.6	208	20.2
Natural menopause	509	49.3	505	49.0
Other surgery	120	11.6	134	13.0
Surgical menopause	160	15.5	154	15.0
Other or missing reasons	30	2.9	29	2.8
**Use of hormone therapy at urine collection**				
Never estrogen (E])	400	38.8	409	39.7
Past E	337	32.7	350	34.0
Current E alone	186	18.0	184	17.9
Current E + progesterone	100	9.7	78	7.6
Missing	9	0.9	9	0.9
**Neighborhood SES at urine collection**				
Quintile 1–low	100	9.7	123	11.9
Quintile 2	147	14.2	153	14.9
Quintile 3	154	14.9	162	15.7
Quintile 4	233	22.6	220	21.4
Quintile 5–high	345	33.4	316	30.7
Missing	53	5.1	56	5.4
**BMI at urine collection (kg/m**^**2**^)				
Mean BMI ± SD	27.1 ± 5.6		26.0 ± 5.6	
< 25	406	39.3	529	51.4
≥ 25- < 30	366	35.5	316	30.7
≥ 30	260	25.2	185	18.0
**Waist-hip ratio (WHR)**^d^				
Mean ± SD	0.862 ± 0.076		0.858 ± 0.082	
< 0.854	384	37.2	475	46.1
≥ 0.854	457	44.3	471	45.7
**Missing**	191	18.5	84	8.2
**Waist (inches)**^d^				
Mean ± SD	35.51 ± 5.51		34.72 ± 5.47	
< 34	331	32.1	425	41.3
≥ 34	513	49.7	524	50.9
**Missing**	188	18.2	81	7.9
**Smoking status**				
Never	578	56.0	618	60.0
Former	315	30.5	300	29.1
Current	133	12.9	104	10.1
Missing	6	0.6	8	0.8
**Mediterranean diet score**^e^				
Quartile 1–low	350	33.9	350	34.0
Quartile 2	207	20.1	204	19.8
Quartile 3	220	21.3	204	19.8
Quartile 4–high	232	22.5	252	24.5
Missing	23	2.2	20	1.9
**Stage**^b^				
In situ	224	21.7		
I (1)	613	59.4		
II (2)	10	1.0		
III (3,4,7)	175	17.0		
Missing	10	1.0		
**Hormone receptor (HR) status**^c^				
Estrogen (ER) + or progesterone (PR)	859	83.2		
ER− and PR−	124	12		
Missing	49	4.7		

^a^All urines from Los Angeles were first morning samples. All urines from Hawaii were overnight (99%) or first morning urines (1%)

^b^Of the 10 missing stage, 2 were ER+ or PR+, 2 were ER− and PR−, and 6 were missing stage

^c^Of the 49 missing HR status, there were 35 in situ, 6 stage I, 1 stage II, 1 stage III, and 6 missing stage

^d^Waist-hip ratio (WHR) information was collected at the third follow-up questionnaire (2003–2006); we included subjects with WHR information before breast cancer diagnosis and for all control women

^e^The Mediterranean diet included 9 components (vegetables, fruit, nuts, legumes, whole grains, fish, alcohol, monounsaturated: saturated fat ratio, and red/processed meat) (Ref [26], Harmon et al.)

The individual phthalate metabolites were modestly correlated (rho’s were 0.2 to 0.4; *P* < 0.05) whereas the DEHP metabolites were more strongly correlated (rho’s were 0.5 to ~ 0.8). The correlations between phthalic acid and the 10 phthalates ranged from 0.15 to 0.44. (Supplementary Table 2).

The risk of breast cancer in all women combined tended to decrease with increasing exposure to MBzP (*P*_trend_ = 0.03). In contrast, risk in all women was positively associated with high ratios of MEHP/MEOHP (*P*_trend_ = 0.04), MEHP/(MEOHP+ MEHHP) (*P*_trend_ = 0.052), and high MEHP% (*P*_trend_ = 0.092); the range of ORs associated with exposure in the upper tertile was 1.18 to 1.26 compared to those in the lowest tertile of exposure. These patterns were apparent among white women (Table 2). Although there were no statistically significant racial/ethnic differences in results, some suggestive differences emerged. Among Native Hawaiian women, risks increased in association with eight of the ten individual phthalates, including MiBP (*P*_trend_ = 0.05), MEHP (*P*_trend_ = 0.01), MEHHP (*P*_trend_ = 0.09), MECPP (*P*_trend_ = 0.06), and phthalic acid (*P*_trend_ = 0.03); ORs ranged from 1.94 to 2.73 for Native Hawaiians in the upper tertile of phthalate exposures compared to those in the lower tertile. In contrast, the individual metabolites were not associated with risk in African Americans and Latinos combined or in Japanese Americans. Among whites, below unity ORs were found for MBzP (*P*_trend_ = 0.05) and for all three DEHP secondary metabolites, with strongest inverse trends for MEOHP (*P*_trend_ = 0.06).

**Table 2:** Associations of breast cancer risk (invasive and in situ) with phthalate metabolites, ratios of DEHP metabolites, and phthalic acid in all women and by race/ethnicity

Metabolites (ng/g creatinine)	All (1032 ca/1030 co)	Whites (274 ca/273 co)	Japanese Americans (478ca/478co)	Native Hawaiians (155 ca/154 co)	African Americans and Latinos (125 ca/125 co)	Phet race^b^
	OR (95% CI)^a^	OR (95% CI)^a^	OR (95% CI)^a^	OR (95% CI)^a^	OR (95% CI)^a^	
**MMP**						
≤ 2.63	1.00	1.00	1.00	1.00	1.00	
> 2.63–7.40	0.82 (0.65–1.04)	0.71 (0.44–1.13)	0.86 (0.61–1.23)	1.16 (0.68–1.99)	0.54 (0.26–1.10)	
> 7.40	0.89 (0.69–1.15)	0.89 (0.53–1.49)	0.84 (0.57–1.24)	1.22 (0.62–2.37)	0.74 (0.36–1.50)	
*P* trend^c^	0.26	0.49	0.36	0.52	0.30	0.59
**MEP**						
≤ 30.64	1.00	1.00	1.00	1.00	1.00	
> 30.64–84.30	1.08 (0.86–1.35)	0.93 (0.60–1.42)	1.06 (0.78–1.45)	1.63 (0.92–2.90)	0.82 (0.33–2.03)	
> 84.30	1.07 (0.84–1.35)	1.13 (0.72–1.78)	0.97 (0.69–1.38)	1.50 (0.85–2.66)	0.75 (0.32–1.80)	
*P* trend^c^	0.56	0.67	0.95	0.12	0.52	0.63
**MBP**						
≤ 12.92	1.00	1.00	1.00	1.00	1.00	
> 12.92–24.60	0.83 (0.66–1.04)	0.99 (0.62–1.58)	0.84 (0.61–1.15)	0.89 (0.51–1.55)	0.53 (0.24–1.16)	
> 24.60	0.87 (0.69–1.11)	0.82 (0.52–1.29)	1.04 (0.73–1.48)	0.64 (0.34–1.20)	0.70 (0.35–1.40)	
P trend^c^	0.21	0.41	0.93	0.20	0.41	0.80
**MiBP**						
≤ 3.12	1.00	1.00	1.00	1.00	1.00	
> 3.12–6.64	0.95 (0.75–1.21)	0.73 (0.44–1.19)	0.86 (0.60–1.22)	1.39 (0.77–2.49)	1.52 (0.68–3.42)	
> 6.64	1.15 (0.89–1.49)	0.74 (0.45–1.23)	1.21 (0.82–1.79)	**2.07 (1.02–4.21)**	1.33 (0.64–2.75)	
*P* trend^c^	0.36	0.23	0.83	**0.05**	0.52	0.16
**MBzP**						
≤ 7.66	1.00	1.00	1.00	1.00	1.00	
> 7.66–15.83	0.81 (0.65–1.01)	0.69 (0.45–1.06)	0.87 (0.63–1.21)	0.67 (0.37–1.21)	1.05 (0.56–1.97)	
> 15.83	**0.79 (0.63–0.99)**	0.67 (0.43–1.03)	0.86 (0.61–1.20)	0.86 (0.47–1.57)	0.77 (0.41–1.46)	
*P* trend^c^	**0.03**	**0.05**	0.35	0.53	0.52	0.56
**MEHP**						
≤ 4.72	1.00	1.00	1.00	1.00	1.00	
> 4.72–10.88	1.00 (0.79–1.26)	0.91 (0.57–1.43)	0.86 (0.61–1.22)	1.74 (0.94–3.22)	1.23 (0.61–2.44)	
> 10.88	1.01 (0.77–1.31)	1.06 (0.46–1.63)	0.76 (0.52–1.12)	**2.73 (1.25–5.97)**	1.08 (0.52–2.27)	
*P* trend^c^	0.97	0.94	0.17	**0.01**	0.81	0.07
**MEHHP**						
≤ 18.44	1.00	1.00	1.00	1.00	1.00	
> 18.44–39.67	0.96 (0.77–1.20)	0.73 (0.47–1.14)	0.98 (0.71–1.34)	1.37 (0.79–2.35)	1.03 (0.53–2.02)	
> 39.67	0.93 (0.74–1.18)	0.69 (0.44–1.11)	0.97 (0.69–1.35)	1.67 (0.90–3.08)	0.80 (0.39–1.61)	
*P* trend^c^	0.56	0.11	0.83	0.09	0.56	0.23
**MEOHP**						
≤ 11.54	1.00	1.00	1.00	1.00	1.00	
> 11.54–26.28	1.03 (0.83–1.28)	0.66 (0.43–1.02)	1.21 (0.88–1.67)	1.21 (0.71–2.05)	1.09 (0.57–2.12)	
> 26.28	0.90 (0.72–1.13)	0.66 (0.42–1.05)	1.03 (0.74–1.44)	1.27 (0.71–2.24)	0.65 (0.33–1.29)	
*P* trend^c^	0.46	0.06	0.67	0.39	0.28	0.24
**MECPP**						
≤ 23.04	1.00	1.00	1.00	1.00	1.00	
> 23.04–45.35	0.94 (0.75–1.18)	0.81 (0.52–1.26)	0.91 (0.65–1.26)	1.22 (0.68–2.19)	1.10 (0.57–2.14)	
> 45.35	0.99 (0.78–1.25)	0.77 (0.49–1.22)	0.98 (0.70–1.38)	**1.94 (1.01–3.70)**	0.80 (0.38–1.66)	
*P* trend^c^	0.84	0.25	0.84	0.06	0.62	0.27
**MCHP**						
≤ 0.34	1.00	1.00	1.00	1.00	1.00	
> 0.34–0.57	1.11 (0.88–1.39)	1.10 (0.71–1.70)	1.19 (0.85–1.67)	1.28 (0.73–2.25)	0.72 (0.36–1.44)	
> 0.57	1.16 (0.91–1.47)	1.25 (0.77–2.03)	1.08 (0.75–1.54)	1.78 (0.91–3.47)	0.86 (0.45–1.64)	
*P* trend^c^	0.23	0.39	0.58	0.10	0.49	0.51
**MEHP%**^**d**^						
≤ 5.49%	1.00	1.00	1.00	1.00	1.00	
> 5.49–11.08%	1.27 (1.00–1.61)	1.46 (0.93–2.30)	1.09 (0.77–1.56)	1.63 (0.88–3.03)	1.22 (0.62–2.38)	
> 11.08%	1.23 (0.94–1.62)	**1.81 (1.06–3.08)**	0.97 (0.65–1.46)	1.46 (0.73–2.92)	1.03 (0.46–2.28)	
*P* trend^c^	0.09	**0.03**	0.94	0.22	0.84	0.44
**MEHP/(MEOHP + MEHHP)**						
≤ 9.76%	1.00	1.00	1.00	1.00	1.00	
> 9.76–21.36%	**1.32 (1.04–1.68**)	1.57 (1.00–2.44)	1.18 (0.82–1.71)	1.46 (0.79–2.70)	1.21 (0.62–2.38)	
> 21.36%	1.26 (0.96–1.66)	**2.10 (1.20–3.65)**	0.98 (0.66–1.47)	1.14 (0.56–2.34)	1.38 (0.63–3.03)	
*P* trend^c^	**0.05**	**0.007**	0.97	0.55	0.42	0.30
**MEHP/(MECCP + MEHHP)**						
≤ 7.87%	1.00	1.00	1.00	1.00	1.00	
> 7.87–16.86%	**1.28 (1.02–1.63**)	1.35 (0.86–2.08)	1.20 (0.85–1.77)	1.38 (0.76–2.49)	1.36 (0.68–2.70)	
> 16.86%	1.18 (0.90–1.56)	**1.76 (1.04–2.99)**	1.01 (0.67–1.53)	1.14 (0.58–2.24)	0.92 (0.41–2.05)	
*P* trend^c^	0.13	**0.04**	0.82	0.56	0.93	0.64
**MEHP/MEHHP**						
≤ 0.159	1.00	1.00	1.00	1.00	1.00	
> 0.159–0.341	1.20 (0.94–1.54)	1.31 (0.84–2.04)	1.07 (0.73–1.57)	1.31 (0.69–2.49)	1.28 (0.64–2.56)	
> 0.341	1.24 (0.94–1.63)	**1.84 (1.05–3.21)**	1.08 (0.72–1.63)	0.90 (0.44–1.84)	1.31 (0.59–2.94)	
*P* trend^c^	0.11	**0.04**	0.70	0.92	0.45	0.60
**MEHP/MEOHP**						
≤ 0.245	1.00	1.00	1.00	1.00	1.00	
> 0.245–0.548	**1.36 (1.08–1.71)**	**1.92 (1.23–2.99)**	1.09 (0.78–1.53)	1.32 (0.73–2.41)	1.48 (0.75–2.93)	
> 0.548	1.26 (0.97–1.63)	**2.02 (1.17–3.50)**	0.92 (0.63–1.35)	1.29 (0.69–2.42)	1.62 (0.75–3.50)	
*P* trend^c^	**0.04**	**0.004**	0.79	0.38	0.20	0.14
**MEHP/MECPP**						
≤ 0.137	1.00	1.00	1.00	1.00	1.00	
> 0.137–0.298	1.17 (0.93–1.48)	1.11 (0.72–1.72)	1.11 (0.78–1.59)	1.36 (0.75–2.47)	1.25 (0.63–2.48)	
> 0.298	0.96 (0.73–1.25)	1.14 (0.69–1.88)	0.80 (0.53–1.21)	1.12 (0.57–2.34)	0.97 (0.43–2.20)	
*P* trend^c^	0.99	0.59	0.78	0.57	0.52	0.78
**Phthalic acid (PA)**						
≤ 36.90	1.00	1.00	1.00	1.00	1.00	
> 36.90–79.76	1.11 (0.88–1.40)	0.95 (0.60–1.52)	1.19 (0.86–1.64)	1.61 (0.81–3.21)	0.81 (0.39–1.69)	
> 79.76	1.21 (0.94–1.56)	0.98 (0.61–1.58)	1.17 (0.80–1.69)	**2.22 (1.08–4.57)**	1.04 (0.47–2.30)	
*P* trend^c^	0.14	0.94	0.32	**0.03**	0.92	0.39

^a^Conditional logistic regression with the matched sets as strata and adjusted for education, number of children, age at menarche, menopausal status, BMI at urine collection, neighborhood socioeconomic status at urine collection, smoking, alcohol intake, and Mediterranean energy adjusted total score. Missing categories of covariates were included in the analyses

^b^*P* heterogeneity (race) df = 4

^c^*P* trend (log phthalate) df = 1

^d^MEHP% was calculated by converting the DEHP metabolites (MEHP, MEHHP, MEOHP, and MECCP) into nanomoles (nmol) using their respective molecular weights (278, 294, 292, and 308 g/mol) and dividing the molar mass of MEHP by the mass of the sum of these metabolites and then multiplying by 100

Breast cancer risk in all women combined was not associated with ∑DEHP and ∑HMWP but exposure to ∑LMWP, ∑LMHMPA, and ∑LMHM_molar_ was positively associated with risk in all women combined, with 18 to 23% higher risk for those in the upper tertile of exposure (Table 3)*.* The positive associations were more prominent among Native Hawaiians who displayed statistically significant or suggestive elevated risks with four of the five summation exposures: ∑LMHMPA (*P*_trend_ = 0.001), ∑LMHM_molar_ (*P*_trend_ = 0.03), ∑LMWP (*P*_trend_ = 0.10), and ∑HMWP (*P*_trend_ = 0.10). Native Hawaiian women in the upper two tertiles of ∑LMHMPA exposure showed a significant 2.4- to 2.6-fold higher risk than their counterparts in the lowest tertile of exposure.

**Table 3:** Associations of breast cancer risk with summary phthalate exposures in all women and by race/ethnicity

Summary phthalate	All (1032 ca/1030 co)	Whites (274 ca/273 co)	Japanese Americans (478 ca/478 co)	Native Hawaiians (155 ca/154 co)	African Americans and Latinos (125 ca/125 co)	Phet race^b^
	OR (95% CI)^a^	OR (95% CI)^a^	OR (95% CI)^a^	OR (95% CI)^a^	OR (95% CI)^a^	
**∑DEHP**^c^						
≤ 63.21	1.00	1.00	1.00	1.00	1.00	
63.21–133.05	1.03 (0.82–1.27)	0.83 (0.54–1.29)	0.99 (0.72–1.36)	1.29 (0.75–2.22)	1.38 (0.71–2.69)	
> 133.05	0.93 (0.73–1.17)	0.74 (0.47–1.18)	0.97 (0.68–1.36)	1.49 (0.79–2.79)	0.76 (0.38–1.52)	
*P* trend^d^	0.61	0.20	0.85	0.21	0.57	0.43
**∑HMWP**^c^						
≤ 78.13	1.00	1.00	1.00	1.00	1.00	
> 78.13–149.24	0.95 (0.77–1.18)	0.73 (0.47–1.14)	0.95 (0.70–1.30)	1.18 (0.68–2.04)	1.29 (0.68–2.43)	
> 149.24	0.99 (0.79–1.25)	0.75 (0.46–1.20)	1.03 (0.74–1.44)	1.76 (0.94–3.29)	0.78 (0.40–1.50)	
*P* trend^d^	0.87	0.18	0.93	0.10	0.62	0.32
**∑LMWP**^c^						
≤ 64.0	1.00	1.00	1.00	1.00	1.00	
> 64.0–145.29	1.15 (0.92–1.44)	1.05 (0.66–1.67)	1.13 (0.82–1.55)	1.38 (0.81–2.38)	0.88 (0.35–2.23)	
> 145.29	1.18 (0.93–1.50)	1.10 (0.68–1.78)	1.24 (0.87–1.77)	1.64 (0.89–3.04)	0.66 (0.29–1.53)	
*P* trend^d^	0.16	0.70	0.23	0.10	0.24	0.44
**∑LMHMPA**^c^						
≤ 226.38	1.00	1.00	1.00	1.00	1.00	
> 226.38–442.34	1.03 (0.83–1.29)	0.87 (0.56–1.36)	0.84 (0.62–1.17)	**2.43 (1.33–4.44)**	1.01 (0.51–1.98)	
> 442.34	1.23 (0.96–1.58)	1.00 (0.62–1.62)	1.19 (0.82–1.73)	**2.66 (1.39–5.12)**	0.73 (0.34–1.57)	
*P* trend^d^	0.15	0.96	0.65	**0.001**	0.47	**0.05**
**∑LMHM**_**molar**_^c^						
≤ 0.581	1.00	1.00	1.00	1.00	1.00	
> 0.581–1.143	1.02 (0.82–1.28)	1.38 (0.87–2.21)	0.70 (0.51–0.97)	1.53 (0.86–2.71)	1.39 (0.68–2.85)	
> 1.143	1.18 (0.92–1.50)	1.33 (0.80–2.20)	1.03 (0.72–1.47)	**1.89 (1.06–3.38)**	0.83 (0.39–1.77)	
*P* trend^d^	0.23	0.25	0.78	**0.03**	0.64	0.18

^a^Conditional logistic regression with the matched sets as strata and adjusted for education, number of children, age at menarche, menopausal status, BMI at urine collection, neighborhood socioeconomic status at urine collection, smoking, alcohol intake, and Mediterranean energy adjusted total score

^b^*P* heterogeneity (race) df = 4

^c^**∑**DEHP, sum of MEHP, MEHHP, MEOHP, and MECPP; **∑**HMWP, sum of high molecular weight phthalates (MBzP, DEHP metabolites, and MECP); **∑**LMWP, sum of low molecular weight phthalates (MMP, MEP, MBP, MiBP); **∑**LMHMPA, sum of **∑**LMWP, **∑**HMWP, and PA; and **∑**LMHM _molar ratios_ were calculated by dividing the concentration of each metabolite by its molecular weight to obtain the molar equivalent (micromoles per liter) and then summed the concentrations in micromoles per liter to get total micromoles per liter of metabolites

^d^*P* trend (log phthalate) df = 1

Results did not suggest statistically significant differences in comparisons between HR+ (*n* = 694) and HR− (*n* = 96) invasive breast cancers in all women combined (Table 4). Risk of HR+ breast was significantly positively associated with ∑LMWP exposure (OR _above vs below median_ = 1.30, 95% CI 1.05–1.60), but this positive association was also observed for HR− breast cancer (OR _above vs below median_ = 1.28, 95% CI 0.81–2.00) (*P*_heterogeneity_ = 0.95). In contrast, the risk of HR− breast cancers was increased in association with phthalic acid exposure (OR _above vs below median_ = 1.59, 95% CI 1.01–2.46) but not HR+ breast cancer (OR _above vs below median_ = 1.02, 95% CI 0.83–1.26) (*P*_heterogeneity_ = 0.08). Above the median exposure to ∑LMHMPA was also associated with HR− breast cancer (OR _above vs below median_ = 1.54, 95% CI 0.98–2.41, *P* = 0.06); this increased risk was observed in whites, Native Hawaiians, and African Americans and Latinos combined, but it was particularly prominent among Native Hawaiian women (OR _above vs below median_ = 4.92, 95% CI 1.33–18.2) (Supplementary Table 3).

**Table 4:** Associations of risk of hormone receptor-positive (HR+) and HR-negative (HR−) invasive breast cancers with summary phthalate exposures in all women combined

Summary phthalate	All women			
	HR+ ***n*** = 694		HR− ***n*** = 96	
	OR (95% CI)^a^	Cases/controls	OR (95% CI)^a^	Cases/controls
**∑DEHP**				
< 118.53	1.00	443/508	1.00	58/508
≥ 118.53	1.02 (0.82–1.26)	251/288	1.05 (0.67–1.64)	38/288
*P* value	0.87		0.83	
**P het**^b^	0.90			
**∑HMWP**				
< 106.79	1.00	369/405	1.00	45/405
≥ 106.79	0.93 (0.76–1.15)	325/391	1.15 (0.75–1.78)	51/391
*P* value	0.50		0.52	
***P*** **het**^b^	0.39			
**∑LMWP**				
< 93.03	1.00	317/408	1.00	40/408
≥ 93.03	**1.30 (1.05–1.60)**	377/388	1.28 (0.81–2.00)	56/388
*P* value	**0.015**		0.29	
***P*** **het**^b^	0.95			
**∑LMHMPA**				
< 310.52	1.00	332/408	1.00	38/408
≥ 310.52	1.17 (0.95–1.44)	362/388	1.54 (0.98–2.41)	58/388
*P* value	0.15		0.06	
***P*** **het**^b^	0.28			
**∑LMHM**_**molar**_				
< 0.80	1.00	331/411	1.00	40/411
≥ 0.80	1.20 (0.98–1.48)	363/385	1.40 (0.90–2.19)	56/385
*P* value	0.08		0.14	
***P*** **het**^b^	0.55			
**Phthalic acid**				
< 53.54	1.00	343/399	1.00	36/399
≥ 53.54	1.02 (0.83–1.26)	351/397	**1.59 (1.01–2.48)**	60/397
*P* value	0.84		**0.04**	
**P het**^b^	0.08			

^a^Unconditional logistic regression adjusting for matching factors including area, age, time of urine collection, type of urine specimen, education, number of children, age at menarche, menopausal status, BMI at urine collection, neighborhood socioeconomic status at urine collection, smoking, alcohol intake, and Mediterranean energy adjusted total score. Both HR+ and HR− were compared to 796 control women in all women combined analyses

^b^*P* heterogeneity (HR+ vs HR−) df = 1

Phthalate-breast cancer associations did not differ by years of follow-up after urine collection, or between all (invasive and in situ combined) versus invasive breast cancers only (data not shown). There were also no suggestive differences in breast cancer associations with phthalate exposures by use of hormone therapy or BMI at urine collection (data not shown). However, in the subgroup of women with data on WHR, breast cancer risks increased among those with high (≥ 0.854) WHR in association with exposure to MEHP%, ratio variables of MEHP to secondary metabolites of DEHP, phthalate acid, and ∑LMHMPA (Table 5). The ORs for ∑LMHMPA were elevated among those with high WHR (OR_T3 vs T1_ = 2.01, 95% CI 1.22–3.32, *P*_trend_ = 0.01) but not among those with low (< 0.854) WHR (OR_T3 vs T1_ = 0.79, 95% CI 0.47–1.31) (*P*_heterogeneity_ = 0.01).

**Table 5:** Associations of breast cancer risk and exposures to DEHP metabolites and summary phthalate exposures by waist-hip ratio (WHR)

	Ca	Co	WHR < 0.854	Ca	Co	WHR ≥ 0.854	Phet (df = 1)^b^
	116	475	OR (95% CI)^a^	117	471	OR (95% CI)^a^	
**MEHP%**^c^							
≤ 5.49%	35	147	1.00	23	170	1.00	
> 5.49–11.08	41	155	1.09 (0.66–1.81)	47	153	**2.29 (1.32–3.98)**	
> 11.08	40	173	0.94 (0.57–1.57)	47	148	**2.39 (1.36–4.18)**	**0.02**
*P* trend			0.86			**0.002**	
**Phthalic acid**							
≤ 36.90	45	143	1.00	30	170	1.00	
> 36.90–≤ 79.76	37	161	0.74 (0.45–1.20)	37	150	1.44 (0.84–2.45)	
> 79.76	34	171	0.64 (0.38–1.07)	50	151	**1.98 (1.18–3.33)**	**0.002**
*P* trend			0.08			**0.01**	
**∑DEHP**							
≤ 63.21	41	149	1.00	39	162	1.00	
63.21–133.05	38	160	0.89 (0.54–1.47)	39	157	1.07 (0.65–1.77)	
> 133.05	37	166	0.90 (0.54–1.50)	39	152	1.16 (0.69–1.93)	0.49
*P* trend			0.66			0.59	
**∑HMWP**							
≤ 78.13	38	147	1.00	41	165	1.00	
> 78.13–149.24	40	159	0.99 (0.60–1.64)	37	156	0.99 (0.60–1.63)	
> 149.24	38	169	0.95 (0.57–1.58)	39	150	1.13 (0.68–1.88)	0.68
*P* trend			0.85			0.69	
**ΣLMWP**							
≤ 64.0	42	154	1.00	37	165	1.00	
> 64.0–145.29	34	144	0.87 (0.53–1.45)	39	162	1.14 (0.69–1.89)	
> 145.29	40	177	0.86 (0.52–1.43)	41	144	1.44 (0.86–2.43)	0.17
*P* trend						0.19	
**∑LMHMPA**							
≤ 226.38	39	136	1.00	35	175	1.00	
> 226.38–442.34	38	157	0.86 (0.52–1.43)	31	157	1.04 (0.61–1.78)	
> 442.34	39	182	0.79 (0.47–1.31)	51	139	**2.01 (1.22–3.32)**	**0.01**
*P* trend			0.35			**0.01**	

Waist-hip ratio (WHR) information was collected at the third follow-up questionnaire (2003–2006); we included subjects with WHR information before breast cancer diagnosis and all control women

^a^Unconditional logistic regression adjusting for matching factors including ethnicity, age, time of urine collection, type of urine specimen, education, number of children, age at menarche, menopausal status, BMI at urine collection, HT use at urine collection, neighborhood socioeconomic status at urine collection, smoking, alcohol intake, and Mediterranean energy adjusted total score

^b^*P* heterogeneity (WHR < 0.854 vs WHR ≥ 0.85) df = 1

^c^MEHP% was calculated by converting the DEHP metabolites (MEHP, MEHHP, MEOHP, and MECPP) into nanomoles (nmol) using their respective molecular weights (278, 294, 292, and 308 g/mol) and dividing the molar mass of MEHP by the mass of the sum of these metabolites and then multiplying by 100

## Discussion

This nested case-control study adds new information on the association of phthalate exposures and breast cancer risk. This is only the second study with data on pre-diagnostic urinary exposures, and the first to examine potential racial/ethnic differences in risk associations in a single study population where similar study methods were used. Although our study still lacked adequate statistical power to detect statistically significant differences in racial/ethnic-specific comparisons, the suggestive risk pattern differences between whites and nonwhites, and between the different groups of nonwhites (Native Hawaiians, African Americans, Latinos, and Japanese Americans), highlight the challenges in studying these ubiquitous chemicals in relation to breast cancer risk. The suggestive differences in associations between HR+ and HR− breast cancer and among those with high WHR warrant confirmation in future studies.

The most consistent findings in all women combined were the inverse associations with MBzP, the increased risks associated with high MEHP%, and high ratios of DEHP metabolites, namely MEHP to MEOHP and MEHHP. The significant inverse association between MBzP exposure and risk is consistent with results observed in Mexico [11] and among white women in the WHI [14] and LIBCSP [13]. While estrogenic effects of BBP, the precursor of MBzP, have been reported in cell culture and other experimental studies [31–33], these estrogenic effects may not be applicable to in vivo environments [34], and anti-estrogenic effects of BBP have been reported in some studies [35].

Our findings on the individual DEHP metabolites and ratios of primary to secondary DEHP metabolites suggest potential racial/ethnic differences in exposure and/or metabolism. Exposures to all four DEHP metabolites were inversely associated with risk in whites but were positively associated with risk in Native Hawaiians; the results in whites are supportive of the null/inverse associations reported previously in whites [13, 14], while the results in Native Hawaiians are broadly consistent with findings among Mexican [11] and Native Alaska women [12]. Risk in all women appeared to be higher in association with high MEHP% and high ratios of MEHP/MEOHP and MEHP/MEHHP. As shown in Fig. 1, DEHP and other high molecular weight long-branch phthalates undergo several biotransformations, including further hydroxylation and oxidation before excretion [36–38]. MEHP, the hydrolysis product of DEHP, is not a major metabolite, while the oxidative metabolites (MEHHP, MEOHP, and MECPP) are the major metabolites of DEHP. As MEHP may be more toxic than the oxidative metabolites, higher MEHP% and higher ratios of MEHP to MEHHP or MEHP to MEOHP may implicate greater physiologic effects as compared with individual metabolites; this seemed to be particularly apparent among women with high WHR. While we are not aware of previous investigations of MEHP% and ratios of DEHP metabolites in relation to breast cancer risk, higher MEHP% and higher MEHP ratios may represent more toxicity in terms of male infertility [29], obesity based on BMI and waist circumferences [30], inflammation, and oxidative stress represented by gamma glutamyltransferase [39]. Oxidative stress-stimulated inflammation may be part of the etiologic pathway for DEHP-induced carcinogenesis.

Our finding between HR+ breast cancer and ∑LMWP is supportive of suggestive effects of phthalates on ER+ breast cancer [32, 40]. In contrast, the risk associations with exposure to phthalic acid, ∑LMHMPA, and ∑LMHM_molar_ appeared to be stronger for HR− than HR+ breast cancers but this was based on only 96 HR− breast cancers. Although these risk associations were particularly strong among Native Hawaiians, we are cautious in our interpretation because of the very wide confidence intervals (Supplementary Table 3). Further investigations of whether HR− breast cancer may be differentially affected by phthalate exposures are needed, as fewer than 300 HR− breast cancers have been investigated in this (*n* = 96) and combined previous studies on urinary phthalates (*n* = 58 in WHI [14] and *n* = 109 in LIBCSP [13]).

Finally, although there was no significant evidence of statistical heterogeneity by race/ethnicity, risk association patterns between phthalate exposures and breast cancer risk were not uniform across the racial/ethnic groups. In particular, risk associations were more prominent among Native Hawaiians; in that group, risk was positively associated with eight of the ten individual phthalate metabolites, phthalic acids, and total phthalates (∑LMHMPA, ∑LMHM_molar_) while the associations were less consistent in the other racial/ethnic groups. We are not aware of previous studies on exposures of phthalates among Native Hawaiians and Japanese Americans in the USA, but the divergent risk patterns may be due to differences in their use of phthalate containing products as suggested by racial/ethnic differences in profiles of individual urinary phthalates (Supplementary Table 1). Variations in genetic susceptibility to metabolize phthalates and other factors affecting metabolism may be also important and warrant investigation.

Strengths of this study include being the first prospective study to investigate urinary phthalate metabolites among five racial/ethnic groups in the same study, and providing the first of such data in Native Hawaiians and Japanese Americans, and carefully considering potential confounders (Supplementary Table 4). We used a highly sensitive assay and carefully examined risk associations with both individual metabolites and all metabolites combined as they may represent different sources and routes of exposure. However, there are several limitations. We relied on a single urine sample measurement; the within-person variability is modest for phthalate metabolites in this study as in other studies [41] and may have reduced the statistical power of our study. Sample sizes of African Americans and Latinas were also modest, which precluded examining their risk patterns separately. In addition, information on pre-diagnostic WHR was available on only a subset of breast cancer cases. Due to the number of tests performed, some of the findings are likely due to chance. All the samples from Los Angeles County were first morning samples, whereas almost all urine specimens from Hawaii were overnight specimens. While misclassification of exposure is unavoidable, we believe that non-differential misclassification of exposure would tend to attenuate the overall results and underestimate the true association.

## Conclusions

Results from this large nested case-control study suggest that exposure to phthalates may be associated with breast cancer risk. However, these relationships are complex and may differ by race/ethnicity and hormone receptor status of breast cancer, perhaps reflecting different exposure patterns to phthalate containing products as well as metabolism. Our novel findings of increased risks with ratios of primary DEHP metabolite (MEHP) to secondary DEHP metabolites (MEHHP, MEOHP, MECPP) emphasize the need of further investigation of not only the individual metabolites, but also ratios of the metabolites as well as summation of these metabolites. While additional studies with pre-diagnostic samples are needed to fill our gaps in understanding the effects of these ubiquitous chemicals, continued efforts to reduce exposure burden to phthalates are needed.

## Supplementary Information

**Additional file 1: Supplement Table 1.** Molecular weight of phthalate metabolites and phthalic acid and samples with lower limit of detection (LLOD), and geometric mean (95%CI) of phthalate metabolites and summary variables of phthalates (μg/g creatinine) by study area and by race/ethnicity. **Supplementary Table 2.** Pairwise Spearman’s correlations between individual phthalate biomarkers and summation variables among controls. **Supplementary Table 3.** Associations of risk (OR and 95% CI) ^**a**^ of hormone receptor positive (HR+) and HR negative (HR-) invasive breast cancers with summary phthalate exposures by race/ethnicity. **Supplemental Table 4.** Distribution of covariates by tertiles of total phthalate exposure (∑LMHMPA).

## Data Availability

Request for additional details of the data used in the manuscript can be directed to the corresponding author.

## Abbreviations

BBP: Butyl benzyl phthalate
BMI: Body mass index kg/m^2^
CI: Confidence interval
CV: Coefficient of variation
DBP: Di-butyl phthalate
DEHP: Di-2-ethylhexyl phthalate
DEP: Di-ethyl phthalate
DMP: Di-methyl phthalate
ER/PR: Estrogen/progesterone receptor
HR+: Hormone receptor-positive status
HR−: Hormone receptor-negative status
HMW: High molecular weight
LLOD: Lower limit of detection
LIBCSP: Long Island Breast Cancer Study Project
LMW: Low molecular weight
MBP: Mono-n-butyl phthalate
MBzP: Mono-benzyl phthalate
MCMHP: Mono 2-carboxy-hexyl phthalate
MEC: Multiethnic Cohort
MECPP: Mono(2ethyl-5-carboxy-pentyl) phthalate
MEHP: Mono-2-ethylhexyl-phthalate
MEHHP: Mono(2-ethyl-5-hydroxyhexyl)
MEOHP: Mono(2-ethyl-5-oxohexy)
MEP: Mono-ethyl phthalate
MMP: Mono-methyl phthalate
MiBP: Mono-isobutyl phthalate
NHANES III: Third National Health and Nutrition Examination Survey
nSES: Neighborhood socioeconomic status
SEER: Surveillance, Epidemiology, and End Results Program
SD: Standard deviation
WHR: Waist-hip ratio
WHI: Women’s Health Initiative
