# Supplementary material for: Urinary phthalate exposures and risk of breast cancer: the Multiethnic Cohort study
Source: Breast Cancer Res. 2021 Apr 6;23:44. doi: 10.1186/s13058-021-01419-6 (PMC8025373; doi:10.1186/s13058-021-01419-6)
Supplement: Supplementary file 1 — Additional file 1: Supplement Table 1. Molecular weight of phthalate metabolites and phthalic acid and samples with lower limit of detection (LLOD), and geometric mean (95%CI) of phthalate metabolites and summary variables of phthalates (μg/g creatinine) by study area and by race/ethnicity. Supplementary Table 2. Pairwise Spearman’s correlations between individual phthalate biomarkers and summation variables among controls. Supplementary Table 3. Associations of risk (OR and 95% CI) a of hormone receptor positive (HR+) and HR negative (HR-) invasive breast cancers with summary phthalate exposures by race/ethnicity. Supplemental Table 4. Distribution of covariates by tertiles of total phthalate exposure (∑LMHMPA). [file 13058_2021_1419_MOESM1_ESM.docx]

Supplement Table 1. Molecular weight of phthalate metabolites and phthalic acid and samples with lower limit of detection (LLOD), and geometric mean (95%CI) of phthalate metabolites and summary variables of phthalates (µg/g creatinine) by study area and by race/ethnicity

|  | |  |  | Geometric mean (95% CI) | | | | | | |
| --- | --- | --- | --- | --- | --- | --- | --- | --- | --- | --- |
|  | |  |  | By Study Area | | By race/ethnicity | | | | |
| Metabolites of phthalate (P) | | Molecular weight (g/mol) | LLOD N  (%) | Overnight  (n=1796, all from Hawaii) | First morning (n=250 from LA, n=6 from Hawaii) | Whites  (n=547)^a^ | Japanese Americans  (n=956) ^a^ | Native Hawaiians  (n=309) ^a^ | African Americans (n=97) ^b^ | Latinos  (n=153) ^b^ |
| MMP | monomethyl P | 180.16 | 405 (19.6%) | 4.0 (3.7,4.3) | 4.9 (4.1,5.8) | 4.6 (4.1,5.2) | 3.9 (3.6,4.3) | 3.4 (2.9,4) | 5.1 (3.5,7.4) | 4.7 (3.7,6.0) |
| MEP | monoethyl P | 194.20 | 11 (0.5%) | 51.9 (49,55.1) | 111.1 (96.8,127.4) | 59.7 (53.1,67.2) | 48.9 (45.2,52.9) | 52.5 (44.8,61.4) | 113.3 (89.6,143.3) | 122.7(101,149) |
| MBP | mono-n-butyl P | 222.30 | 17 (0.8%) | 17.6 (17,18.3) | 25.8 (22.9,29) | 20.1 (18.6,21.7) | 16.8(15.8,17.8) | 16.3(14.8,18) | 23.6(19.4,28.7) | 29.4 (24.6,35.0) |
| MiBP | monoisobutyl P | 222.30 | 152 (7.4%) | 4.4 (4.2,4.7) | 6 (5.3,6.7) | 5.3 (4.8,5.9) | 4.2(3.9,4.6) | 4(3.6,4.5) | 5.9(4.7,7.3) | 6.1 (5.2,7.1) |
| MBzP | monobenzyl P | 256.25 | 65 (3.2%) | 10.8 (10.4,11.2) | 7.1 (6.3,8.0) | 11.0 (10.2,11.9) | 10.8(10.2,11.5) | 9.7(8.6,10.9) | 6.6(5.2,8.3) | 7.2 (6.1,8.6) |
| MEHP | mono-2-ethylhexyl P | 278.34 | 135 (6.5%) | 7.2 (6.8,7.6) | 6.4 (5.5, 7.3) | 7.5 (6.8,8.2) | 7.2(6.7,7.8) | 6.2(5.3,7.3) | 5.1(4,6.5) | 7.2 (6.1,8.6) |
| MEHHP | mono-(2-ethyl-5-hydroxyhexyl) P | 294.34 | 16 (0.8%) | 30.6 (28.8,32.4) | 27.9 (24.8, 31.4) | 35.5 (32.2,39.2) | 28.8 (26.6,31.1) | 27.7 (24.1,31.7) | 22.9 (19.2,27.3) | 33.4 (28.6,39.1) |
| MEOHP | mono-(2-ethyl-5-oxohexyl) P | 292.33 | 24 (1.2%) | 19.3 (18.2,20.5) | 17.5 (15.5,19.6) | 23.1 (21.4,25.0) | 17.8 (16.8,18.9) | 16.8 (14.6,19.2) | 14.0 (11.5,17.0) | 20.7 (17.4, 24.7) |
| MECPP | mono-(2-ethyl-5-carboxy-pentyl) P | 308.33 | 7 (0.3%) | 37.0 (35.5,38.4) | 33.8 (30.0, 38.0) | 38.9 (35.9,42.0) | 36.2 (34.2,38.4) | 34.8 (31.0,39.2) | 23.8 (20.4,27.8) | 42.9 (36.7,50.2) |
| MCHP | mono-cyclohexyl P | 248.27 | 232 (11.3%) | 0.5 (0.5,0.5) | 0.4 (0.3,0.4) | 0.5 (0.5,0.5) | 0.5(0.5,0.5) | 0.5(0.4,0.5) | 0.4(0.3,0.4) | 0.4(0.4,0.5) |
| PA | phthalic acid | 166.13 | 7 (0.3%) | 63.4 (59.8,67.3) | 82.3(71.7,94.4) | 79.0 (71.7,87.2) | 55.1 (51.0, 59.6) | 65.4 (57.0, 75.0) | 81.5 (67.0, 99.1) | 87.4 (73.2,104.2) |
|  | ∑LMWP ^c^ |  |  | 101 (97,105) | 178 (158,200) | 118 (109,128) | 95 (89,100) | 99 (88,111) | 178 (146, 216) | 196 (165,234) |
|  | ∑HMWP ^c^ |  |  | 124 (119, 129) | 106 (96,117) | 136 (125,147) | 120 (113,128) | 112 (102,124) | 83 (71, 97) | 128 (111,149) |
|  | ∑LMHMPA ^c^ |  |  | 354 (341, 368) | 441 (400,487) | 408 (377, 441) | 334 (315, 354) | 344 (306,387) | 395 (338, 463) | 503 (430, 588) |

^a^ Study subjects from Hawaii
^b^ Study subjects from Los Angeles County
**^c^** **∑**LMWP- sum of low molecular weight phthalates (MMP, MEP, MBP, MiBP); **∑**HMWP- sum of high molecular weight phthalates (MBzP, **∑**DEHP – sum of MEHP, MEHHP, MEOHP, MECPP; and MCHP),
**∑**LMHMPA-sum of **∑**LMWP, **∑**HMWP, and PA

Supplementary Table 2. Pairwise Spearman’s correlations between individual phthalate biomarkers and summation variables among controls

|  | MMP | MEP | MBP | MIBP | MBzP | MEHP | MEHHP | MEOHP | MECPP | MCHP | PA | ∑DEHP | ∑LMWP | ∑HMWP | ∑LMHMPA | ∑LMHM_MOLAR_ |
| --- | --- | --- | --- | --- | --- | --- | --- | --- | --- | --- | --- | --- | --- | --- | --- | --- |
| MEP | 0.25 | 1.00 |  |  |  |  |  |  |  |  |  |  |  |  |  |  |
| MBP | 0.21 | 0.31 | 1.00 |  |  |  |  |  |  |  |  |  |  |  |  |  |
| MIBP | 0.24 | 0.26 | 0.43 | 1.00 |  |  |  |  |  |  |  |  |  |  |  |  |
| MBZP | 0.17 | 0.12 | 0.33 | 0.23 | 1.00 |  |  |  |  |  |  |  |  |  |  |  |
| MEHP | 0.32 | 0.18 | 0.24 | 0.27 | 0.21 | 1.00 |  |  |  |  |  |  |  |  |  |  |
| MEHHP | 0.20 | 0.2 | 0.26 | 0.22 | 0.22 | 0.5 | 1.00 |  |  |  |  |  |  |  |  |  |
| MEOHP | 0.20 | 0.22 | 0.31 | 0.25 | 0.25 | 0.53 | 0.84 | 1.00 |  |  |  |  |  |  |  |  |
| MECPP | 0.18 | 0.18 | 0.24 | 0.21 | 0.20 | 0.45 | 0.86 | 0.77 | 1.00 |  |  |  |  |  |  |  |
| MCHP | 0.21 | 0.11 | 0.12 | 0.16 | 0.15 | 0.29 | 0.06^a^ | 0.12 | 0.06 | 1.00 |  |  |  |  |  |  |
| PA | 0.38 | 0.44 | 0.30 | 0.21 | 0.21 | 0.37 | 0.32 | 0.36 | 0.29 | 0.15 | 1.00 |  |  |  |  |  |
| ∑DEHP | 0.24 | 0.21 | 0.26 | 0.24 | 0.23 | 0.6 | 0.92 | 0.88 | 0.92 | 0.12 | 0.36 | 1.00 |  |  |  |  |
| ∑LMWP | 0.41 | 0.91 | 0.52 | 0.39 | 0.20 | 0.26 | 0.25 | 0.28 | 0.22 | 0.16 | 0.51 | 0.26 | 1.00 |  |  |  |
| ∑HMWP | 0.24 | 0.22 | 0.30 | 0.25 | 0.38 | 0.59 | 0.89 | 0.86 | 0.89 | 0.13 | 0.37 | 0.97 | 0.27 | 1.00 |  |  |
| ∑LMHMPA | 0.43 | 0.61 | 0.42 | 0.30 | 0.30 | 0.52 | 0.61 | 0.61 | 0.59 | 0.16 | 0.78 | 0.68 | 0.70 | 0.70 | 1.00 |  |
| ∑LMHM_MOLAR_ | 0.39 | 0.75 | 0.44 | 0.34 | 0.30 | 0.46 | 0.61 | 0.59 | 0.59 | 0.16 | 0.57 | 0.66 | 0.83 | 0.67 | 0.92 | 1.00 |

^a^ All p values <0.05 except for MCHP and MEHHP

≥≥≥

Supplementary Table 3. Associations of risk (OR and 95% CI) **^a^** of hormone receptor positive (HR+) and HR negative (HR-) invasive breast cancers with summary phthalate exposures by race/ethnicity

|  | **Whites** | | **Japanese Americans** | | **Native Hawaiians** | | **African Americans and Latinos** | |
| --- | --- | --- | --- | --- | --- | --- | --- | --- |
|  | **HR+ n= 191** | **HR- n= 26** | **HR+ n=320** | **HR- n= 32** | **HR+ n=112** | **HR- n=13** | **HR+ n=71** | **HR- n=25** |
|  | OR(95%CI) (Ca/Co) | OR(95%CI) (Ca/Co) | OR(95%CI) (Ca/Co) | OR(95%CI) (Ca/Co) | OR(95%CI) (Ca/Co) | OR(95%CI) (Ca/Co) | OR(95%CI) (Ca/Co) | OR(95%CI) (Ca/Co) |
| **∑DEHP** |  |  |  |  |  |  |  |  |
| <118.53 | 1.00 (114/128) | 1.00 (15/128) | 1.00 (209/236) | 1.00 (24/236) | 1.00 (73/85) | 1.00 (7/85) | 1.00 (47/59) | 1.00 (12/59) |
| ≥118.53 | 1.02 (77/89)  (0.68-1.52) | 0.99 (11/89)  (0.43-2.28) | 1.05 (111/119)  (0.76-1.44) | 0.58 (8/119)  (0.24-1.36) | 1.13 (39/40)  (0.65-1.95) | 1.79 (6/40)  (0.56-5.79) | 0.78 (24/40)  (0.41-1.51) | 1.71 (13/40)  (0.67-4.34) |
| P value | 0.94 | 0.98 | 0.79 | 0.21 | 0.67 | 0.89 | 0.47 | 0.26 |
| P het ^b^ | 0.95 |  | 0.20 |  | 0.48 |  | 0.18 |  |
| **∑HMWP** |  |  |  |  |  |  |  |  |
| <106.79 | 1.00 (103/102) | 1.00 (14/102) | 1.00 (172/217) | 1.00 (19/217) | 1.00 (51/69) | 1.00 (4/69) | 1.00 (18/28) | 1.00 (5/28) |
| ≥106.79 | 0.80 (88/115)  (0.53-1.19) | 0.74 (12/115)  (0.32-1.69) | 0.94 (148/138)  (0.69-1.28) | 0.91 (13/138)  (0.43-1.92) | 1.21 (61/56)  (0.72-2.02) | 1.32 (9/56)  (0.42-4.17) | 0.87 (53/71)  (0.46-1.65) | 2.53 (20/71)  (0.95-6.73) |
| P value | 0.26 | 0.47 | 0.72 | 0.80 | 0.47 | 0.64 | 0.67 | **0.06** |
| P het ^b^ | 0.87 |  | 0.93 |  | 0.90 |  | **0.07** |  |
| **∑LMWP** |  |  |  |  |  |  |  |  |
| <93.03 | 1.00 (76/94) | 1.00 (12/94) | 1.00 (172/217) | 1.00 (19/217) | 1.00 (51/69) | 1.00 (4/69) | 1.00 (18/28) | 1.00 (5/28) |
| ≥93.03 | 1.16 (115/123)  (0.78-1.73) | 0.85 (14/123)  (0.37-1.94) | 1.35 (148/138)  (0.99-1.85) | 1.24 (13/138)  (0.58-2.65) | 1.46 (61/56)  (0.87-2.46) | 2.62 (9/56)  (0.76-9.08) | 1.20 (53/71)  (0.59-2.44) | 1.73 (20/71)  (0.58-5.20) |
| P value | 0.45 | 0.69 | **0.056** | 0.59 | 0.15 | 0.13 | 0.61 | 0.33 |
| P het ^b^ | 0.50 |  | 0.83 |  | 0.40 |  | 0.59 |  |
| **∑LMHMPA** |  |  |  |  |  |  |  |  |
| <310.52 | 1.00 (80/90) | 1.00 (9/90) | 1.00 (170/205) | 1.00 (18/205) | 1.00 (50/77) | 1.00 (4/77) | 1.00 (32/36) | 1.00 (7/36) |
| ≥310.52 | 1.03 (111/127)  (0.69-1.53) | 1.46 (17/127)  (0.60-3.54) | 1.17 (150/150)  (0.86-1.59) | 1.07 (14/150)  (0.51-2.25) | **1.99** (62/48)  **(1.18-3.35)** | **4.92** (9/48)  **(1.33-18.15)** | 0.74 (39/63)  (0.39-1.39) | 1.57 (18/63)  (0.58-4.24) |
| P value | 0.90 | 0.40 | 0.31 | 0.85 | **0.01** | **0.02** | 0.35 | 0.37 |
| P het ^b^ | 0.48 |  | 0.83 |  | 0.21 |  | 0.21 |  |
| **∑LMHM_molar_** |  |  |  |  |  |  |  |  |
| <0.80 | 1.00 (82/97) | 1.00 (10/97) | 1.00 (164/202) | 1.00 (18/202) | 1.00 (54/81) | 1.00 (5/81) | 1.00 (31/31) | 1.00 (7/31) |
| ≥0.80 | 1.12 (109/120)  (0.76-1.67) | 1.36 (16/120)  (0.58-3.17) | 1.23 (156/153)  (0.90-1.67) | 1.08 (14/153)  (0.51-2.27) | **2.00** (58/44)  **(1.18-3.40)** | **3.72** (8/44)  **(1.09-12.71)** | 0.61 (40/68)  (0.32-1.16) | 1.21 (18/68)  (0.44-3.33) |
| P value | 0.56 | 0.48 | 0.19 | 0.84 | **0.01** | **0.04** | 0.13 | 0.72 |
| P het ^b^ | 0.69 |  | 0.75 |  | 0.37 |  | 0.27 |  |
| Phthalic acid |  |  |  |  |  |  |  |  |
| <53.54 | 1.00 (78/90) | 1.00 (9/90) | 1.00 (181/202) | 1.00 (16/202) | 1.00 (51/67) | 1.00 (4/67) | 1.00 (33/40) | 1.00 (7/40) |
| ≥53.54 | 1.04 (113/127)  (0.70-1.56) | 1.37 (17/127)  (0.56-3.35) | 0.96 (139/153)  (0.71-1.31) | 1.32 (16/153)  (0.64-2.74) | 1.49 (61/58)  (0.88-2.52) | 2.84 (9/58)  (0.82-9.83) | 0.75 (38/59)  (0.40-1.40) | 1.74 (18/59)  (0.66-4.60) |
| P value | 0.84 | 0.50 | 0.82 | 0.46 | 0.13 | 0.10 | 0.36 | 0.26 |
| P het ^b^ | 0.59 |  | 0.44 |  | 0.35 |  | 0.15 |  |

**^a^** Unconditional logistic regression adjusting for matching factors including area, age, time of urine collection, type of urine specimen, education, number of children, age at menarche, menopausal status, BMI at urine collection, neighborhood socioeconomic status at urine collection, smoking, alcohol intake, and Mediterranean energy adjusted total score. Both HR+ and HR- were compared to 796 control women in all women combined analyses.
**^b^** P trend (log phthalate) df=1
**^c^** P heterogeneity (HR+vs HR-) df=1

Supplemental Table 4. Distribution of covariates by tertiles of total phthalate exposure (∑LMHMPA)

|  | Controls | | Tertile 1 ∑LMHMPA | | Tertile 2 ∑LMHMPA | | Tertile 3  ∑LMHMPA | |
| --- | --- | --- | --- | --- | --- | --- | --- | --- |
|  | N=1030 | % | N=341 | % | N=339 | % | N=350 | % |
| **Area** |  |  |  |  |  |  |  |  |
| Hawaii | 881 | 85.5 | 311 | 91.2 | 291 | 85.8 | 279 | 79.7 |
| Los Angeles | 149 | 14.5 | 30 | 8.8 | 48 | 14.2 | 71 | 20.3 |
| **Urine type ^a^** |  |  |  |  |  |  |  |  |
| First morning | 157 | 15.2 | 35 | 10.3 | 48 | 14.2 | 74 | 21.1 |
| Overnight | 873 | 84.8 | 306 | 89.7 | 291 | 85.8 | 276 | 78.9 |
| **Mean age at urine collection, yrs+SD** | 66.3 ±7.8 | | 66.4 ±7.9 | | 66.1 ±8.0 | | 66.4 ±7.4 | |
| ≤64 | 470 | 45.6 | 147 | 43.1 | 163 | 48.1 | 160 | 45.7 |
| 65-74 | 387 | 37.6 | 140 | 41.1 | 114 | 33.6 | 133 | 38.0 |
| 75+ | 173 | 16.8 | 54 | 15.8 | 62 | 18.3 | 57 | 16.3 |
| **Race/ethnicity** |  |  |  |  |  |  |  |  |
| Japanese American | 478 | 46.4 | 176 | 51.6 | 162 | 47.8 | 140 | 40.0 |
| White | 273 | 26.5 | 71 | 20.8 | 97 | 28.6 | 105 | 30.0 |
| Native Hawaiian (NH) | 154 | 15.0 | 71 | 20.8 | 40 | 11.8 | 43 | 12.3 |
| African American (AA) | 49 | 4.8 | 12 | 3.5 | 15 | 4.4 | 22 | 6.3 |
| Latino | 76 | 7.4 | 11 | 3.2 | 25 | 7.4 | 40 | 11.4 |
| **Education** |  |  |  |  |  |  |  |  |
| ≤High school | 424 | 41.2 | 144 | 42.2 | 136 | 40.1 | 144 | 41.1 |
| Some college | 217 | 21.1 | 69 | 20.2 | 74 | 21.8 | 74 | 21.1 |
| College graduate | 202 | 19.6 | 74 | 21.7 | 64 | 18.9 | 64 | 18.3 |
| Graduate school | 180 | 17.5 | 52 | 15.2 | 63 | 18.6 | 65 | 18.6 |
| Missing | 7 | 0.7 | 2 | 0.6 | 2 | 0.6 | 3 | 0.9 |
| **Age at menarche, yrs** |  |  |  |  |  |  |  |  |
| <12 | 570 | 55.3 | 177 | 51.9 | 204 | 60.2 | 189 | 54.0 |
| 13-14 | 359 | 34.9 | 131 | 38.4 | 97 | 28.6 | 131 | 37.4 |
| >14 | 95 | 9.2 | 30 | 8.8 | 37 | 10.9 | 28 | 8.0 |
| Missing | 6 | 0.6 | 3 | 0.9 | 1 | 0.3 | 2 | 0.6 |
| **Number of Children** |  |  |  |  |  |  |  |  |
| Nulliparous | 111 | 10.8 | 35 | 10.3 | 40 | 11.8 | 36 | 10.3 |
| 1 child | 116 | 11.3 | 33 | 9.7 | 36 | 10.6 | 47 | 13.4 |
| 2-3 children | 553 | 53.7 | 181 | 53.1 | 191 | 56.3 | 181 | 51.7 |
| >4 children | 245 | 23.8 | 89 | 26.1 | 71 | 20.9 | 85 | 24.3 |
| Missing | 5 | 0.5 | 3 | 0.9 | 1 | 0.3 | 1 | 0.3 |
| **Age at first live birth, yrs** |  |  |  |  |  |  |  |  |
| Nulliparous | 111 | 10.8 | 35 | 10.3 | 40 | 11.8 | 36 | 10.3 |
| 15-20 | 208 | 20.2 | 70 | 20.5 | 64 | 18.9 | 74 | 21.1 |
| 21-30 | 614 | 59.6 | 206 | 60.4 | 199 | 58.7 | 209 | 59.7 |
| >30 | 76 | 7.4 | 23 | 6.7 | 31 | 9.1 | 22 | 6.3 |
| Missing | 21 | 2.0 | 7 | 2.1 | 5 | 1.5 | 9 | 2.6 |
| **Menopausal status** |  |  |  |  |  |  |  |  |
| Premenopause | 208 | 20.2 | 72 | 21.1 | 59 | 17.4 | 77 | 22.0 |
| Natural menopause | 505 | 49 | 167 | 49 | 162 | 47.8 | 176 | 50.3 |
| Other surgery | 134 | 13 | 33 | 9.7 | 53 | 15.6 | 48 | 13.7 |
| Surgical menopause | 154 | 15 | 60 | 17.6 | 54 | 15.9 | 40 | 11.4 |
| Other or missing reasons | 29 | 2.8 | 9 | 2.6 | 11 | 3.2 | 9 | 2.6 |
| **Use of Hormone therapy at urine collection** | | |  |  |  |  |  |  |
| Never Estrogen [E] | 409 | 39.7 | 133 | 39.0 | 140 | 41.3 | 136 | 38.9 |
| Past E | 350 | 34.0 | 123 | 36.1 | 117 | 34.5 | 110 | 31.4 |
| Current E alone | 184 | 17.9 | 50 | 14.7 | 63 | 18.6 | 71 | 20.3 |
| Current E + Progesterone | 78 | 7.6 | 30 | 8.8 | 19 | 5.6 | 29 | 8.3 |
| missing | 9 | 0.9 | 5 | 1.5 | 0 | 0 | 4 | 1.1 |
| **Neighborhood SES at urine collection** | |  |  |  |  |  |  |  |
| Quintile 1-low | 123 | 11.9 | 39 | 11.4 | 38 | 11.2 | 46 | 13.1 |
| Quintile 2 | 153 | 14.9 | 55 | 16.1 | 57 | 16.8 | 41 | 11.7 |
| Quintile 3 | 162 | 15.7 | 54 | 15.8 | 49 | 14.5 | 59 | 16.9 |
| Quintile 4 | 220 | 21.4 | 74 | 21.7 | 71 | 20.9 | 75 | 21.4 |
| Quintile 5-high | 316 | 30.7 | 102 | 29.9 | 103 | 30.4 | 111 | 31.7 |
| Missing | 56 | 5.4 | 17 | 5.0 | 21 | 6.2 | 18 | 5.1 |
| **BMI at urine collection (kg/m^2^**) | |  |  |  |  |  |  |  |
| Mean BMI ± SD | 26.0 ± 5.6 | | 26.2 ± 6.1 | | 25.8 ± 5.6 | | 25.9 ± 5.1 |  |
| <25 | 529 | 51.4 | 178 | 52.2 | 177 | 52.2 | 174 | 49.7 |
| 25-<30 | 316 | 30.7 | 101 | 29.6 | 104 | 30.7 | 111 | 31.7 |
| 30 | 185 | 18.0 | 62 | 18.2 | 58 | 17.1 | 65 | 18.6 |
| **Waist hip ratio (WHR)^b^** |  |  |  |  |  |  |  |  |
| Mean ± SD | 0.86 ±0.08 | | 0.87 ±0.08 | | 0.86 ±0.08 | | 0.85 ± 0.08 | |
| <0.854 | 475 | 46.1 | 136 | 39.9 | 157 | 46.3 | 182 | 52.0 |
| ≥0.854 | 471 | 45.7 | 175 | 51.3 | 157 | 46.3 | 139 | 39.7 |
| Missing | 84 | 8.2 | 30 | 8.8 | 25 | 7.4 | 29 | 8.3 |
| **Waist (inches) ^b^** |  |  |  |  |  |  |  |  |
| Mean ± SD | 34.7 ± 5.5 | | 35.1 ± 5.5 | | 34.6 ± 5.5 | | 34.4 ± 5.4 | |
| <34 | 425 | 41.3 | 136 | 39.9 | 138 | 40.7 | 151 | 43.1 |
| ≥34 | 524 | 50.9 | 176 | 51.6 | 178 | 52.5 | 170 | 48.6 |
| **missing** | 81 | 7.9 | 29 | 8.5 | 23 | 6.8 | 29 | 8.3 |
| **Smoking status** |  |  |  |  |  |  |  |  |
| Never | 618 | 60.0 | 204 | 59.8 | 206 | 60.8 | 208 | 59.4 |
| Former | 300 | 29.1 | 93 | 27.3 | 98 | 28.9 | 109 | 31.1 |
| Current | 104 | 10.1 | 40 | 11.7 | 33 | 9.7 | 31 | 8.9 |
| Missing | 8 | 0.8 | 4 | 1.2 | 2 | 0.6 | 2 | 0.6 |
| **Mediterranean diet score ^c^** | | |  |  |  |  |  |  |
| Quartile 1 –low | 350 | 34 | 114 | 33.4 | 119 | 35.1 | 117 | 33.4 |
| Quartile 2 | 204 | 19.8 | 72 | 21.1 | 64 | 18.9 | 68 | 19.4 |
| Quartile 3 | 204 | 19.8 | 64 | 18.8 | 71 | 20.9 | 69 | 19.7 |
| Quartile 4-high | 252 | 24.5 | 84 | 24.6 | 78 | 23 | 90 | 25.7 |
| Missing | 20 | 1.9 | 7 | 2.1 | 7 | 2.1 | 6 | 1.7 |

^a^ All urines from Los Angeles were first morning samples. All urines from Hawaii were overnight (99%) or first morning urines (1%).
^b^ Waist hip ratio(WHR) information was collected at the third follow-up questionnaire (2003-2006); we included subjects with WHR information for all control women.
^c^ The Mediterranean diet included 9 components (vegetables, fruit, nuts, legumes, whole grains, fish, alcohol, monounsaturated: saturated fat ratio, and red/processed meat) (Ref 26, Harmon et al., 2015)
